# Age-Tastic! An Evaluation of an Evidence-Based Intervention for Older Adults

**DOI:** 10.3390/healthcare6040142

**Published:** 2018-12-08

**Authors:** Manoj Pardasani, Jackie Berman, Mebane Powell

**Affiliations:** 1 Silberman School of Social Work, Hunter College, 695 Park Avenue, New York, NY 10065, USA; 2 New York City Department for the Aging, New York, NY 10007, USA; jberman@aging.nyc.gov (J.B.); mpowell@aging.nyc.gov (M.P.)

**Keywords:** Evidence-based, senior centers, health promotion, game theory, randomized control trial

## Abstract

*Background*: Raising awareness of holistic health and safety among older adults is critical to enhancing their wellbeing in many cases, improving health outcomes and motivating positive behavioral changes. Age-Tastic! is a comprehensive health and safety promotion intervention that uses the concept of a competitive board game to entice older adults to participate and stay engaged. *Objective*: The purpose of this study was to evaluate the impact of Age-Tastic! on the level of awareness, health literacy, self-efficacy and positive behavioral change among the participants. *Methods*: A randomized control trial was conducted with 98 older adults assigned to an experimental and control group. Interviews were conducted at baseline, right after the eight-week intervention ended and again eight weeks after the end of the intervention. *Results*: The results showed significant increases among experimental group participants in knowledge about health, self-efficacy and behavioral change in the areas of nutrition, financial exploitation, health literacy and emotional well-being. *Discussion*: Implications for replication and engagement are discussed.

## 1. Introduction

The Older Americans Act (OAA) of 1965 helped establish the widest network of community-based services for older adults in the US. OAA helped fund diverse programs such as senior centers, congregate and home-delivered meals, access and transportation, case management, elder abuse prevention and caregiver support. One critical component of the OAA is Title III-D which focused on disease prevention and health promotion for community-dwelling older adults. In 2016, the Administration for Community Living (ACL) which oversees the Administration of Aging, announced that only evidence-based programs would be funded under Title III-D. These programs are mainly to be offered by community-based organizations such as senior centers. These organizations around the country have now been faced with the decision to use or possibly lose Title III-D funding if they do not implement evidence-based health promotion programming at their sites. While evidence-based programming serves the purpose of providing programs known to have a beneficial impact to seniors’ health and wellness, they are oftentimes expensive to implement. The lack of resources and financial support for senior centers to implement these programs is a major challenge. Furthermore, most of the evidence-based programs offered in community settings tend to focus on one aspect of health—physical health, chronic disease, nutrition, falls prevention or mental health. It is rare to find an intervention that specifically addresses multiple aspects of physical, emotional and mental health, as well as personal safety in healthful behaviors in one comprehensive intervention. Age-Tastic! is one such intervention that was designed to address that gap.

### 1.1. The Changing Demographics of Older Adults in New York City

According to the U.S. Census Bureau population estimates, adults ages 65 and over grew from 35.0 million in 2000 to 49.2 million in 2016, accounting for 15.2% of the total population. This cohort is projected to more than double to over 98 million by 2060 [1]. In New York City, individuals age 60 and older comprise 18.6% of the population, or 1.56 million adults. The city’s older adult population is expected to increase to 1.86 million by 2040, comprising 20.6% of the total population. One in five (21%) of NYC’s older adults also live in poverty. Underscoring this is the fact that minority older adults are at greater risk of poverty—29% of Latino/a, 22% of Asian Americans, and 19% of African Americans/Blacks live in poverty. Less than half of the older adults (43.1%) have achieved an educational level beyond a High School diploma, thereby limiting their income potential. Nearly, one in three older adults (28.9%) lives alone [2]. Based on the New York City Community Health Survey (2016), over one-third of older adults, 65 years and older report their health as fair or poor (39.9%), while close to one in ten report they are currently suffering from depression (9.1%), over one-fourth have diabetes (27.5%), close to one-third had not exercised in the past 30 days (33.0%), 6.6% suffer from financial exploitation and 9.1% report never consuming fruit or vegetables [2,3]. However, 15.9% consume on average 1 or more sugar-sweetened beverage daily [4].

Poor health, low health literacy, falling, becoming a victim of a financial exploitation, suffering from mental illness and poor nutrition are all risk factors for increased disability, social isolation, and early institutionalization. Data collected within New York City indicate that falls are the leading cause of injury-related death and hospitalizations for older adults 65 and older, with one in three older adults falling each year, resulting in 29,000 emergency department visits, 17,000 hospitalizations, and 250 deaths [5]. The Federal Trade Commission (2017), one out of 5 seniors fall victim to fraud, which accounts for over 300,000 older adults in New York City alone [6]. The number of adults aged 65 and older who suffer from mental illness in New York City was projected to increase from 495,000 in 2000 to 772,000 in 2030 [7]. According to a report by Hunger Free America, between 2013 and 2016, an average of 13.6% of New Yorkers over the age of 60 lived in food insecure households, approximately 30% more than between 2006 and 2008 [8] (p. 12). Finally, when it comes to health literacy and awareness, close to one-third of older adults have below what is considered basic levels of health literacy, which represents over 500,000 older adults in New York City [9].

### 1.2. Barriers to Implementation of Current Evidence-Based Health Promotion Interventions

In addition to challenges of implementation, fidelity and sustainability, most evidence-based programs that have been implemented in senior centers (A Matter of Balance, Chronic Disease Self-Management Program, Wisdom Works, PEARLS, etc.) focus on one specific issue like memory retention, mental concentration, falls prevention or chronic disease management. A holistic health and wellness program that incorporates all aspects of health (social, physical, mental, financial and cultural) has not been implemented until recently. The illness-specific programs may not be attractive to a wider audience as they may not think of themselves as needing help in one of these areas. Also, the perceived stigma of participation in such programs may deter some older adults. Similarly, with mental illness and substance abuse, older adults often do not seek out evidence-based programming or any such programming due to the fear of being associated with the illness and/or lack of ability to recognize that they may be experiencing these issues [10,11,12,13]. Also, many of these programs are structured as learning opportunities and may not be viewed as a “fun” activity by the intended targets of these programs.

### 1.3. Behavioral Activation, Cognitive Structuring and Game Theory

Behavioral activation theory has served as the building block of many evidence-based programs developed to address mental health issues in older adults and is also one of the central components of the prevention program, ‘A Matter of Balance’ [14], as well as diabetes self-management programs. Researchers have found that any intervention that reinforces learning through discussion, application (trial activities) and positive reinforcement ultimately enhances the motivation and skills needed for change [15]. In addition to behavioral activation, evidence-based programs often include components of cognitive-behavior therapy, such as cognitive restructuring. Cognitive restructuring techniques “include challenging the truthfulness of a thought by looking at evidence for and against the thought, identifying thinking errors the thought exemplifies, and developing alternative thought that more realistically reflect their experience” [16] (p. 454).

Kapp (2012), a renowned researcher in game theory, underscores the importance of keeping the player engaged and tailoring the game to the interests of your audience [17]. Concepts of game theory include reward systems (motivation to participate), chance components (generating excitement) and competition (promotes social interaction). Research has found that games that incorporate these aspects of game theory are more likely to continue to appeal to the game player and that participants are more likely to learn and retain when compared to traditional teaching methods [17] (p. 138). Therefore, using game theory as the foundational base for the intervention and integrating the concepts of behavioral activation and cognitive structuring in to the intervention, one can create an impactful and engaging health intervention such as Age-Tastic! The concepts of game theory, chance, competition and rewards are embedded in the very design of the intervention, as well as its implementation. Age-Tastic! is a board game where participants roll a die and land on a specific category (chance), have to answer questions in that category in a limited time (competition) and earn game “dollars” for every correct answer (reward). At the end of the game, the participant with the highest number of dollars wins a prize. More detail on the intervention is provided in Section 2.2 below.

## 2. Materials and Methods

### 2.1. Design

This study employed a longitudinal, randomized control trial (RCT) design to evaluate the impact of the intervention on the knowledge, self-efficacy and behavioral change among the participants. Study participants were recruited from six senior centers in New York City through convenience sampling. We made presentations at the senior centers about the intervention and invited members to sign up. They were informed that they might be in a group that actually got to participate in the intervention or a group that would not participate. Those who could not participate were assured that the intervention would be offered again after six months and they would get preference for participation at that time. The older adults who signed up were randomly assigned to the experimental (intervention) or control group. Those assigned to the experimental group participated in the Age-Tastic! program which lasted eight weeks. Those in the control group were assigned to a wait list if they indicated an interest in participation in the intervention. They did not receive any alternative intervention. All study participants were interviewed at three time points—baseline (prior to the start of the intervention), eight (8) weeks (after the end of the intervention) and at sixteen (16) weeks (eight weeks after the end of the intervention).

### 2.2. Intervention—Age-Tastic!

In 2013, the NYC Department for the Aging (DFTA) received a community innovations grant from the Administration of Community Living (ACL) to develop an innovative Health and Wellness game for older adults. During the course of the grant, the game was developed, tested and refined by health and wellness experts, as well as an advisory team of older adults. At the conclusion of the grant, DFTA developed the evidence-based program, Age-Tastic!. The developers of the game incorporated the concepts of behavioral activation, cognitive restructuring and game theory into the design of this intervention to make it appealing and engaging for older adults. The intervention focuses on health literacy and promotion in a holistic manner incorporating subjects such as physical and mental health, knowledge of aging processes, financial and environmental safety, and nutrition. Through the use of game play, group discussions and home activities this program aims to build health literacy about critical components of physical and emotional health, nutrition, social support, physical and financial safety; enhance self-efficacy and motivation for health and well-being; and engage older adults to engage in behavioral change through the development of skills in the areas of communication with providers, building social networks, preventing fraud and abuse, self-advocacy, falls prevention, healthy eating and engagement in healthful behaviors (behavioral change).

Once the program was developed, it was piloted in the community with older senior center participants. In total, the program and materials were tested four times before being implemented into the community. By 2017, NYC DFTA partnered with Fordham University’s Graduate School of Social Service, to conduct a randomized control trial in hopes of documenting the programs ability to be classified as an evidence-based program.

The Age-Tastic! program is played in eight modules of one-hour each and can be implemented ideally with 6–15 senior participants per intervention. Modules were created to include emotional wellbeing (Feeling ‘Alright), physical fitness and falls prevention (Keep it moving), fraud protection and financial mistreatment (Money Matters), nutrition (Food for thought) and other health promotion topics such as chronic disease management and benefits and entitlements for older adults (Mix it Up). The Age-Tastic! program comes as a board game similar to *Trivial Pursuit* (a popular board game with various categories of questions). However, the addition to this game is the inclusion of game money which participants earn as they answer questions correctly. The board game comes with 100 cards per category (such as well-being, fitness, nutrition, fraud prevention, healthcare). The board game includes cards (with questions on one side of the card and answers on the other side of the card), a die, a timer, a facilitator’s guide outlining all the steps of the program and discussion questions. Each module starts with game play, which involves each player rolling a die to indicate which of the main topic areas they would be asked to answer, with the aim of gaining an Age-Tastic! dollar for a correct answer in a limited time. After this initial round of game play, players participate in the “Lightning Round” of game play where participants are introduced to a particular topic (e.g., falls) which corresponds to a pre-selected set of 10 cards that focus on some aspect of that topic (e.g., wearing proper footwear, taping down rugs). Once the ten cards are exhausted, the moderator leads a facilitated discussion of the topic of the week (e.g., should you call your doctor if you fall; why are older adults more prone to falling; how can one prevent falls inside and outside the home, etc.). This is then followed by a “Try It At Home” activity that reinforces the day’s discussion (e.g., falls prevention checklist) through homework. Each module concludes with participants tallying up their dollars and leaving with the intent to complete the “Try It At Home” activity. The following week includes a report back from the players about the homework activity, followed by a debriefing where key points are reinforced (e.g., using grab bars, having good lighting), followed by a new round of game play and a new “Lightning Round” topic, discussion topic and “Try It At Home” activity. Each week a different “lighting round” topic area is discussed. Winners of the program are disclosed at the 8th session, where participants can then decide whether they would like to select a moderator and continuing the game play version of Age-Tastic! and, thus, continuing to promote concepts used within the 8-week sessions.

The program resources needed to train the facilitators (board game, manual, on-site or virtual training, video support materials) are cost effective and older adult volunteers could be trained to lead this intervention. At the time of this study, the New York City Department for the Aging (DFTA) paid for the actual board games which were then used in the senior centers. The cost of the board game is relatively low (under $100 US dollars) and can be easily purchased by senior centers and other aging providers. But in addition to the purchase of the game, resources are needed to train the facilitators in the facilities where the game will be played. It is hoped that after one or two rounds of implementation, older adult volunteers could be trained to help facilitate the intervention.

### 2.3. Sample

A total of 105 older adults started with the study. However, at the end of the study, we had completed all three interviews with 98 participants. There were 64 participants in the experimental group and 34 participants in the control group. Both the groups were representative of the general senior center population characteristics. A large proportion of the sample was female (72%). Approximately two in three participants were Caucasian (63.5%), one in five were African American/Black (20.4%), followed by Latino/a (14.3%). Nearly 7% of the sample identified as “Other” and only 2% of the sample identified as Asian American. There were no significant differences in gender or ethnicity between the experimental and control group participants. The gender and ethnicity distribution of the sample closely resembled the senior center participant profile. One in ten participants (11.2%) reported being married or living with a significant other. Over half (58.2%) were separated, widowed or divorced and 26.5% were never married. One fourth of the sample had an education up to a High school diploma (24.4%), while 32.6% had completed an associate degree or some college. One in five participants had completed their Bachelor’s degree (20.4%) while an equal proportion reported earning a Master’s degree (21.4%). There were no significant differences between the experimental and control group participants with respect to living status or education.

### 2.4. Data Collection

Approval by the Institutional Review Board (IRB) at Fordham University was obtained prior to the start of the study. Announcements about the intervention were made at six senior centers located in New York City. Potential participants were informed about the Age-Tastic! intervention, as well as the evaluation. Interested participants were asked to provide names and contact information. The interested participants were then randomly assigned to the experimental and control groups. They were then contacted by telephone to inform them of their status and asked for their permission to participate in the evaluation study.

All willing participants were interviewed by phone at all three time points (baseline, eight weeks and sixteen weeks). Interviews were conducted by trained research assistants. For the purpose of this evaluation, all interviews were conducted in English only. Participants were paid $20 for each interview and the payments were mailed to them after the completion of each interview. The names and contact information was maintained by the Principal Investigator (PI) until the interviews had been concluded. After the completion of interviews, all identifying information was deleted from the data set and office records.

### 2.5. Measures

The interview questionnaire was drafted by the PI in collaboration with the co-authors of this manuscript. The instrument contained three standardized instruments including a screening for depression (PHQ-9), Self-rated Mental Health (SRMH comprising one item) and the Self-rated Physical Health (SRH—one item). All other items were crafted by the authors of this study and assessed:

(i) Participant demographics (age, ethnicity, living status, income, etc.).
(ii) Perception of aging.
(iii) Experience with nutrition labels on food packages.
(iv) Frequency and nature of contact with medical providers.
(v) Awareness of personal medication regimens.
(vi) Current chronic health conditions and use of assistive devices.
(vii) Perception of self-efficacy (managing one’s life) and social support.
(viii) Experience with falls and prevention efforts.
(ix) Knowledge of financial fraud prevention.
(x) Experience with the intervention.*
(xi) Engagement in change (healthful) behaviors as a result of participating in the intervention.* (* these were only asked of the participants who completed the intervention.)

### 2.6. Protocol Administration

The baseline interviews took approximately 45 min to administer, while the follow-up interviews took approximately thirty (30) min. Participants could choose not to answer any specific questions or discontinue the interviews altogether. All interviews were conducted via telephone at a time scheduled by the participant.

### 2.7. Data Analysis

The data were analyzed using SPSS (Statistical Package for Social Sciences). Univariate analyses were conducted to describe the study sample. Bivariate and multivariate analyses were conducted, including chi square tests to evaluate the impact of the intervention on the participants. When appropriate, Fischer’s Exact Test was also used to test for significant relationships.

## 3. Results

### 3.1. Differences between Experimental and Control Group at Baseline (Prior to Start of the Intervention)

We evaluated the differences between the experimental and control group in three domains:

- Knowledge of critical issues related to aging.
- Perception of self-efficacy.
- Behavioral change (engagement with healthful behaviors).

We found no statistically significant differences between the two groups on any items in the three domains.

### 3.2. Differences between Experimental and Control Group at 8 Weeks (End of Intervention)

#### 3.2.1. Increases in Knowledge of Critical Issues Related to Aging

We found significant differences in increased knowledge around topics concerning health literacy, financial exploitation and emotional wellbeing (Table 1). When asked about their awareness of issues related to aging, participants in the experimental group were more likely to agree (desired response) with the following statements than the control group participants:

- It is important to tell your doctor about all the over-the counter medications, vitamins, and herbal supplements you take.
- If you think you have been a victim of fraud, you should contact your bank.

Participants in the experimental group were more likely to disagree (desired response) with the following statements than the control group participants.

- Depression is a normal part of getting older.
- If you have friends to talk to about your problems, you don’t need to see a therapist/counselor.
- Giving out your social security number on the phone is fine, as long as the person calling you seems trustworthy.

The differences between the experimental and control group participants in the items above were statistically significant.

#### 3.2.2. Self-Efficacy (Change in Attitudes)

Significant differences were found in increased self-efficacy in the areas of nutrition and emotional well-being among the experimental group participants. With regards to the importance of healthful behaviors, participants in the experimental group were more likely to perceive the importance of those behaviors than their control group counterparts (Table 2). The statistically significant items were:

- Read the nutritional label on food packages (*p* = 0.005 Fischer’s Exact Test).
- Regularly socialize with friends (*p* = 0.01 Fischer’s Exact Test).
- Try new social activities (*p* = 0.03 Fischer’s Exact Test).

#### 3.2.3. Behavioral Change

We found significant improvements among experimental group participants in assertive communication with medical providers, engaging in physical activity to improve health, and the ability to prevent financial exploitation.

Participants in the experimental group were more likely to bring a list of questions for their doctor and ask detailed questions regarding their health (Table 3). Both items had statistically significant differences between the experimental and control groups. With respect to the frequency of engaging in healthful behaviors, participants in the experimental group engaged with greater frequency than their control group participants in the following activities (Table 4):

- Exercise (*p* = 0.02 Fischer’s Exact Test).
- Read the nutritional label on food packages (*p* = 0.005 Fischer’s Exact Test).
- Change your personal identification number (PIN) regularly (*p* = 0.01 Fischer’s Exact Test).

### 3.3. Differences between Experimental and Control Group Participants at 16 Weeks (8 Weeks after the End of the Intervention)

We interviewed all the participants eight (8) weeks after the end of the intervention. In our evaluation we looked at those items that had statistically significant differences between the experimental and control groups at the end of the intervention.

#### 3.3.1. Increases in Knowledge of Critical Issues Related to Aging

We continued to find significant differences in increased knowledge around topics concerning health literacy, financial exploitation and emotional wellbeing. We found the following items to continue to have statistically significant differences between the two groups with participants from the experimental group reporting a heightened awareness of issues related to aging in contrast to their control group counterparts:

- It is important to tell your doctor about all the over-the counter medications, vitamins, and herbal supplements you take (*p* = 0.04).
- Depression is a normal part of getting older (*p* = 0.000).
- If you have friends to talk to about your problems, you don’t need to see a therapist/counselor (*p* = 0.001).
- Giving out your social security number on the phone is fine, as long as the person calling you seems trustworthy (*p* = 0.000).

#### 3.3.2. Self-Efficacy (Change in Attitudes)

Significant differences were found in increased self–efficacy in the areas of physical health (fitness) and nutrition. We did not find a continued change in attitudes towards socialization between the two groups at this time phase. With regards to the importance of healthful behaviors, participants in the experimental group continued to perceive the importance of those behaviors as more important than their control group counterparts. The statistically significant items were:

- Exercise (*p* = 0.000).
- Read the nutritional label on food packages (*p* = 0.000).

#### 3.3.3. Behavioral Change

We continued to find significant improvements among experimental group participants in assertive communication with medical providers, engaging physical activity to improve health, and nutrition. The engagement in activity to avoid financial exploitation (like changing the PIN on your bank and credit cards) did not continue to be significantly different for the two groups.

Participants in the experimental group were more likely to report that they continue to bring a list of questions for their doctor (*p* = 0.001) and ask detailed questions regarding their health (*p* = 0.001). With respect to the frequency of engaging in healthful behaviors, participants in the experimental group continued to engage with greater frequency than their control group participants in the following activities:

- Exercise (*p* = 0.000).
- Read the nutritional label on food packages (*p* = 0.000).

### 3.4. Overall Impact of Participation in Age-Tastic!

An overwhelming majority of participants in the experimental group (94.3%) rated their satisfaction with the intervention as “Good” to “Excellent”. Over three out of four participants (79.3%) stated that they learned “somewhat to a great deal” from the intervention. Nine out of ten (94%) participants stated they would recommend the intervention to a friend.

With respect to changes made in one’s life as a result of the intervention, 61.5% of the participants stated they had made changes to prevent financial exploitation, reduce the risk of falls, watching nutritional intake and being an active participant during medical visits. With respect to the specific changes, a large proportion of participants stated they made “somewhat or a great deal” of changes in identifying fall hazards (76.5%), evaluating different activities engaged in (82.4%), identifying ways to protect oneself from being a victim of identity theft (76%) or a scam (70%), reading food labels (88.1%) making healthy choices in eating (87.7%) and making the most out of a doctor’s visit (83.7%).

## 4. Discussion

The Age-Tastic! program’s aim was to improve the health and wellbeing of older adults participating in the intervention. Lifestyle changes often do not happen in a vacuum. First, older adults need to better understand what is needed to improve their health and wellness (knowledge gain); second, they must feel that they can effect change (improved self-efficacy), which finally leads to making lifestyle changes, which can improve one’s health.

### 4.1. Knowledge and Awareness

Compared to the control group participants, the experimental group participants showed significant gains in knowledge around physical fitness, mental health, nutrition, financial fraud, and socialization. Health literacy with respect to communication with medical providers also improved significantly for this cohort. The vast majority of these increases in knowledge and awareness were maintained at 16 weeks. Participants were also asked to rate the importance of making behavioral changes with respect to health, physical activity, socialization, nutrition, environmental (preventing falls) and financial safety. Compared to the control group participants, the experimental group participants were significantly more likely to perceive the importance of health behaviors, especially within areas of nutrition, exercise, and socialization. Most of these gains were also maintained at 16 weeks by the experimental group cohort. 

### 4.2. Self-Efficacy and Behavioral Change 

Compared to the control group, the experimental group took more ownership in their health behaviors, through bringing lists of questions to discuss with their provider and having detailed discussions about health concerns with them. Participants in the experimental group also were significantly more likely to make nutritional changes, such as reading food labels to make healthy food choices and exercising. Although not statistically significant, the experimental group participants were more likely to increase the extent to which they socialized with family and friends and tried new social activities in an attempt to stave off the ills of social isolation. Moreover, older adults were more likely to be mindful of being cognizant of financial safety precaution. Almost all of these gains were maintained at 16 weeks.

### 4.3. Overall Impact of the Intervention

Finally, participants were asked about their views and satisfaction with the program. Virtually all of the participants were satisfied, the vast majority felt that they learned a great deal from the program and would recommend it to friends. When asked if they had made changes in the lifestyles due to their participation in the game, the approximately two out of three participants stated they had made changes in identifying fall hazards, evaluating different activities engaged in, identifying ways to protect oneself from being a victim of identity theft or a scam, reading food labels making healthy choices in eating and making the most out of a doctor’s visit to some extent or the other.

We believe that a critical contribution of this study is to provide support to the idea that a holistic health and safety intervention aimed at older adults can be effective and yield positive results in knowledge and health behavioral change. The integration of a game element that is played in groups makes it exciting, enticing and keeps the participants engaged. Furthermore, this is a relatively inexpensive intervention that can be introduced in a vast array of community-based settings such as senior centers, naturally occurring retirement centers (NORCs), assisted living facilities, recreation clubs, congregate meal sites, faith—based socialization groups and even skilled nursing facilities. The resources needed to train the facilitators are cost effective and older adult volunteers could be trained to lead this intervention. The authors feel (based on informal feedback from the facilitators) that the combination of game play, group discussions and homework activities made learning fun and reinforced the understanding/knowledge for the participants.

One of the limitations of this study is the sample size. The final number of participants was 98. We believe that this study should be replicated in other senior centers or community-locations and continue to be evaluated.

In some areas there was no significant difference between the knowledge and behaviors of participants in the experimental and control groups. We believe that the source of study sample may have impacted these results. All our participants are members of senior centers where health promotion and safety workshops are offered generally but with varying frequency and of uneven quality standards. So, members of both groups may already have had some basic knowledge of these issues being taught through Age-Tastic! Perhaps, this intervention should be replicated across different types of venues with diverse participants, which may yield additional positive gains for the participants in Age-Tastic!

## 5. Conclusions

Greater attention is being paid to improving or maintaining the health of older adults in order to reduce expensive medical costs, reduce the frequency of hospitalizations and exorbitant nursing home expenditures. But on a humanistic note, maintaining good health allows an older adult to live with dignity, be integrated within their community, enhance socialization and engagement and improve their quality of life. Interventions that are engaging, attractive and cost effective can make a difference in the lives of older adults. They improve knowledge, awareness, self-efficacy and motivation to engage in healthful and safe behaviors. This intervention makes a small but critical contribution towards this endeavor.

## Figures and Tables

**Table 1:** Perceptions of aging at 8 weeks (after end of intervention).

Perception Items	Responses	Control %	Experimental %	χ^2^	df	*p*
Depression is a normal part of aging	Disagree	55.9	80.6	6.66	1	0.01 *
	Agree	44.1	19.4			
	Total	100.0	100.0			
Physical activity can improve your mood	Disagree	16.1	4.8	3.45	1	0.06
	Agree	83.9	95.2			
	Total	100.0	100.0			
If you have friends to talk about your problems, you don’t need a therapist	Disagree	53.1	85.7	11.94	1	0.001 *
	Agree	46.9	14.3			
	Total	100.0	100.0			
It is important to tell your doctor about all of the over-the-counter medications, vitamins and supplements	Disagree	17.6	0.0			0.001 **
	Agree	82.4	100.0			
	Total	100.0	100.0			
The only thing you need to look for on a nutrition label is the calories	Disagree	85.3	95.3	2.97	1	0.08
	Agree	14.7	4.7			
	Total	100.0	100.0			
On food labels, the ingredients are listed in order by how much is in the food	Disagree	16.7	16.0	0.01	1	0.94
	Agree	83.3	84.0			
	Total	100.0	100.0			
If you think you have been a victim of fraud, you should contact your bank	Disagree	14.7	1.6			0.02 **
	Agree	85.3	98.4			
	Total	100.0	100.0			
If you ask someone close to you for help taking care of your money, they are free to spend what they want	Disagree	88.2	93.7	0.86	1	0.36
	Agree	11.8	6.3			
	Total	100.0	100.0			
Giving out your social security number on the phone is fine	Disagree	87.5	100.0			0.01 **
	Agree	12.5	0.0			
	Total	100.0	100.0			
Staying home will reduce your chances of falling	Disagree	82.4	91.9	1.98	1	0.16
	Agree	17.6	8.1			
	Total	100.0	100.0			
Throw rugs and clutter on the floor are possible dangers that may lead to falls	Disagree	5.9	1.6	1.36	1	0.24
	Agree	94.1	98.4			
	Total	100.0	100.0			
If you feel healthy, you don’t have to go the doctor for—up	Disagree	79.4	90.5	2.33	1	0.13
	Agree	20.6	9.5			
	Total	100.0	100.0			

* Chi Square *p* < 0.05, ** Fischer’s Exact *p* < 0.05.

**Table 2:** Healthful Behaviors: How important is it to you to….

Healthful Behaviors	Importance	Control %	Experimental %	χ^2^	df	*p*
Exercise	Somewhat important	23.5	9.4	2.72	1	0.11
	Extremely important	76.5	90.6			
	Total	100.0	100.0			
Limit foods high in sugar	Not important at all	2.9	3.1	4.59	2	0.1
	Somewhat important	32.4	14.1			
	Extremely important	64.7	82.8			
	Total	100.0	100.0			
Limit foods that are high in cholesterol, sodium and fat	Not important at all	0.0	1.6	3.0	2	0.22
	Somewhat important	29.4	15.6			
	Extremely important	70.6	82.8			
	Total	100.0	100.0			
Read the nutritional label on food packages	Not important at all	11.9	0.9	4.69	2	0.005 *
	Somewhat important	23.5	12.5			
	Extremely important	64.7	87.5			
	Total	100.0	100.0			
Inspect your home for dangers that may increase your risk of falling	Not important at all	0.0	1.6	0.78	2	0.68
	Somewhat important	14.7	11.1			
	Extremely important	85.3	87.3			
	Total	100.0	100.0			
Actively try to do things to reduce the risk of falls	Not important at all	8.8	14.1	2.1	2	0.36
	Somewhat important	38.2	25.0			
	Extremely important	52.9	60.9			
	Total	100.0	100.0			
Regularly socialize with friends and/or family	Not important at all	11.8	0.0	9.04	2	0.01
	Somewhat important	20.6	14.1			
	Extremely important	67.6	85.9			
	Total	100.0	100.0			
Engage in social activities outside the home	Not important at all	11.8	4.7	5.36	2	0.07
	Somewhat important	26.5	12.5			
	Extremely important	61.8	82.8			
	Total	100.0	100.0			
Try new social activities	Not important at all	17.6	4.7	6.81	2	0.03 *
	Somewhat important	35.3	25.0			
	Extremely important	47.1	70.3			
	Total	100.0	100.0			
Check your bank and credit card statements	Not important at all	5.9	6.3	0.31	2	0.86
	Somewhat important	23.5	18.8			
	Extremely important	70.6	75.0			
	Total	100.0	100.0			
Change your PIN	Not important at all	29.0	24.2	0.44	2	0.8
	Somewhat important	32.3	38.7			
	Extremely important	38.7	37.1			
	Total	100.0	100.0			

* Chi Square *p* < 0.05.

**Table 3:** Communication with Medical Providers at 8 weeks (after the end of intervention).

Items	Frequency	Control %	Experimental %	χ^2^	df	*p*
Bring with you a list of questions to ask your doctor	Sometimes	56.7	28.9	5.32	1	0.02 *
	Always	43.3	71.1			
	Total	100.0	100.0			
Ask questions about your health and well-being	Sometimes	43.8	17.9	5.62	1	0.02 *
	Always	56.3	82.1			
	Total	100.0	100.0			
Discuss emotional issues	Sometimes	73.7	90.3	2.43	1	0.12
	Always	26.3	9.7			
	Total	100.0	100.0			
Show your doctor a list of medications, vitamins and/or supplements	Sometimes	50.0	62.2	0.38	1	0.38
	Always	50.0	37.8			
	Total	100.0	100.0			
Discuss medications, vitamins and/or supplements you take	Sometimes	39.3	32.5	0.33	1	0.56
	Always	60.7	67.5			
	Total	100.0	100.0			

* Chi Square *p* < 0.05.

**Table 4:** Healthful Behaviors: How often do you…

Healthful Behaviors	Frequency	Control %	Experimental %	χ^2^	df	*p*
Exercise	Never or rarely	17.6	1.6	8.75	2	0.01 *
	Sometimes	14.7	18.4			
	Often	67.6	74.5			
	Total	100.0	100.0			
Limit foods that are high in sugar	Never or rarely	8.8	6.3	0.34	2	0.84
	Sometimes	35.3	32.8			
	Often	55.9	60.9			
	Total	100.0	100.0			
Limit foods that are high in cholesterol, sodium and fat	Never or rarely	0.0	6.3	2.3	2	0.32
	Sometimes	42.4	35.9			
	Often	57.6	57.8			
	Total	100.0	100.0			
Read the nutritional label on food packages	Never or rarely	18.2	1.7	10.61	2	0.005 *
	Sometimes	24.2	15.0			
	Often	57.6	83.3			
	Total	100.0	100.0			
Actively try to do things that to reduce falls	Never or rarely	26.5	23.4	0.11	2	0.94
	Sometimes	23.5	25.0			
	Often	50.0	51.6			
	Total	100.0	100.0			
Regularly socialize with friends	Never or rarely	5.9	4.7	1.33	2	0.52
	Sometimes	26.5	17.2			
	Often	67.6	78.1			
	Total	100.00	100.0			
Engage in social activities outside the home	Never or rarely	5.9	4.7	0.82	2	0.66
	Sometimes	29.4	21.9			
	Often	64.7	73.4			
	Total	100.0	100.0			
Try new social activities	Never or rarely	14.7	10.9	2.3	2	0.32
	Sometimes	47.1	45.3			
	Often	38.2	43.8			
	Total	100.0	100.0			
Check your bank and credit card statements	Never or rarely	5.9	9.4	1.25	2	0.53
	Sometimes	26.5	34.4			
	Often	67.6	56.3			
	Total	100.0	100.0			
Change your PIN	Never or rarely	75.0	50.8	6.1	2	0.03 *
	Sometimes	25.0	47.5			
	Often	0.0	3.3			
	Total	100.0	100.0			

* Chi Square *p* < 0.05.

## Information on Age-Tastic!

For more information on how to obtain the Age-Tastic! program, please contact the developers at the New York City Department for the Aging at agetastic@aging.nyc.gov.
